# List of Cases Treated in the Surgical Wards of the Pennsylvania Hospital, and Discharged between December 12th and December 31st, 1838

**Published:** 1839-01-05

**Authors:** T. Harris

**Affiliations:** Attending Surgeon


### CLINICAL REPORTS.

### PENNSYLVANIA HOSPITAL.

*List of Cases treated in the Surgical Wards of the Pennsylvania Hospital, and discharged between December 12th and December 31st*, 1838. Dr. T. Harris, Attending Surgeon.

[Reported by H. H. Smith, M. D., Resident Surgeon.]

A case of contusion of the left side of the chest, and of the thigh, hand, and leg of the same side, caused by a fall from a scaffold, was admitted November 28th; treated by cups to the chest, frictions to the other parts, and perfect rest: discharged December 15th, seventeen days after admission. A slight pleurisy followed the blow on the chest, but was relieved in a short time by free depletion and small doses of antimony.

A case of concussion of the brain, caused by a fall through a hatchway, from the fourth story of a warehouse, was admitted November 26th. The man was insensible at the time of his admission; extremities cold, and pulse slow and feeble. Sinapisms were placed over the stomach, upon the extremities; the head shaved, and cold applied to it. In the course of six hours the pulse rose, when the man was bled sixteen ounces, and afterwards purged. After the reaction from the first bleeding, he was cupped to the back of the head, and on the third day he was entirely conscious. Discharged cured, December 15th, nineteen days after the accident.

A case of fracture of the sixth, seventh, and eighth ribs, with a contusion of the knee joint, caused by a fall, was admitted October 27th. A tight roller was applied around the chest, the limb was placed at rest in a long fracture box, and freely leeched. Pleurisy followed the injury to the chest; was treated accordingly, and the man discharged December 19th, fifty-four days after the accident. In this case, the injury to the knee proved more serious than that to the chest. Violent inflammation came on, about three days after the man's entrance, which was overcome only by repeated leechings, fomentations, and perfect rest.

A case of contusion of the back of the hand, caused by the falling of a weight on it, was admitted October 17th. A slight wound existed, which was drawn together by adhesive strips. The arm and hand were then loosely bandaged on a splint, and leadwater constantly applied. Erysipelas, however, attacked it, and the whole back, and a portion of the palm sloughed. This was treated by poultices, and gentle motion every second or third day, until cicatrization was complete, by which any permanent contraction of the fingers, or stiffness of the wrist, was prevented; and the patient was discharged with a perfect hand, December 19th, sixty-three days after the accident.

A case of fracture of the middle of the radius and ulna, was admitted November 13th; two splints, well padded on the sides next to the arm, and extending from the elbow beyond the fingers, were loosely applied, and the arm placed in a sling. This dressing was continued until the patient's discharge, on the 19th of December, motion being occasionally made at the joints of the fingers, wrist, and elbow, to prevent stiffness.

A case of fracture of the leg, was admitted October 20th, the limb was placed in a fracture box, and an evaporating lotion applied to diminish the inflammation. A portion, however, sloughed, rendering the fracture compound. The patient's recovery was consequently not completed until the sixty-ninth day after the accident. This case exemplifies the advantage of the treatment by the fracture box in use in the hospital; the limb being simply placed on a pillow covered with oil-cloth, and the sides of the box drawn up, any application can readily be made without at all moving the parts, which cannot be the case where the limb is surrounded by a roller.

A case of fracture of the right thigh, at the middle, and at the lower third; of the left thigh, at the upper third, together with concussion of the brain, caused by an injury from a locomotive, was admitted December 24th; death took place December 26th.

A case of fracture of the skull, with considerable depression of the bone, caused by blasting, was admitted December 19th, four hours after the injury. The man was comatose, but with a feeble pulse, and had been a hard drinker. After removing the hair, an incision was made on the scalp, over the occipital bone, and a piece an inch and a half square drawn out of the brain by Dr. Harris. Near an ounce of cerebral matter followed its removal; the wound was drawn together and dressed with lint, and the next day poulticed. The man was sensible, sat up in bed and answered questions readily; pulse slow, regular, and weak: ordered tinct. valerian. f.ʒj, every two hours. This state of things continued for four days, the man being sensible, complaining of no pain, and demanding no treatment, but occasional purges of calomel, &c. On the fifth day there was no union of the wound, but some suppuration, with indications of hernia cerebri, the protrusion being the size of a walnut. It continued to increase, notwithstanding pressure was used to some extent. Delirium ensued, with sloughing of the protruding mass, and on the ninth day he died. An examination after death was refused, and the opportunity lost of comparing the case with two preceding cases of hernia cerebri, lately in the hospital, in which large abscesses were found in the brain, near the seat of injury.

A case of fracture of the leg, of contusion of the other leg and ankle, and of a lacerated wound of the thigh, caused by a rail-road car passing over the patient, was admitted December 24th; mortification followed, and the man sank rapidly, dying five days after admission.

